# Editorial: Immunological regulation to enteroviruses and respiratory viruses: infection and vaccination responses

**DOI:** 10.3389/fimmu.2026.1797986

**Published:** 2026-02-24

**Authors:** Huiwen Zheng

**Affiliations:** Institute of Medical Biology, Chinese Academy of Medical Sciences and Peking Union Medical College, Kunming, China

**Keywords:** enterovirus, immunity, immunoregulation, pathogenesis, respiratory virus, vaccination

This Research Topic integrates reviews, pathogenesis findings, and clinical studies to offer a comprehensive assessment of recent advances in viral immunology and vaccine development. From the analysis of neutralizing antibodies against non-polio enteroviruses to the persistence of long-term immune responses following COVID-19 vaccination, these contributions highlight the complexity of host–pathogen interactions and the promise of future antiviral and vaccine strategies.

Four review articles provide an integrated perspective on antiviral immunity and vaccine innovation. Wang et al. synthesize current knowledge on neutralizing antibody responses against major non−polio enteroviruses (NPEVs), including EV−A71, CVA16, EV−D68, and echoviruses. It outlines key mechanisms underlying antibody efficacy, such as interference with receptor binding, internalization, and uncoating, and discusses the application prospects of bispecific or multivalent antibodies. These structural and mechanistic insights provide a scientific foundation for the rational design of future antiviral therapeutics (Frontiers | Neutralizing antibody landscape of the non-polio Enteroviruses and future strategy*).* Zhou et al. highlight Coxsackievirus A6 (CVA6) vaccine development across diverse platforms, including inactivated, virus−like particle (VLP), and subunit vaccines. Comparative animal data reveal substantial variation in immunogenicity, protective efficacy, and cross−reactive responses among candidates. Given the frequent recombination events of CVA6 and the continuous evolution of its pathogen spectrum, the authors emphasize the importance of developing multivalent and mRNA vaccines to better control emerging dominant serotypes (Frontiers | Recent advances on coxsackievirus A6 vaccine research). Alandijany et al. systematically evaluated the efficacy, safety, and immunogenicity of the three FDA-;approved RSV vaccines—Arexvy, Abrysvo, and mResvia. Collectively, these vaccines are capable of providing sustained immune protection with favorable safety profiles in high-;risk populations, thereby offering crucial evidence for global RSV prevention and control strategies. (Frontiers | Evaluating the efficacy, safety, and immunogenicity of FDA-approved RSV vaccines: a systematic review of Arexvy, Abrysvo, and mResvia). Hartnell et al. present a comprehensive review that explores the dynamic changes in Type I interferon (T1IFN) signaling across the human lifespan. They highlight how these shifts shape population-level susceptibility to viral infections. By examining the evolution of immune responses throughout life, the authors provide a theoretical framework for developing individualized treatment strategies, fostering more inclusive research designs, and ultimately improving outcomes for both the youngest and oldest patients. (Frontiers | What goes up must come down: dynamics of type 1 interferon signaling across the lifespan). Together, these reviews illustrate how antibody−mediated protection, vaccine design, and innate immune regulation converge to shape host defense against diverse viral pathogens.

Beyond the review articles, several studies in this Research Topic expand our understanding of viral and bacterial pathogenesis. These include molecular epidemiology of adenoviruses in children, cellular-level analysis of SARS-CoV-2 subgenomic RNAs, and the use of animal models such as C57BL/6J mice for *Mycobacterium abscessus* infection and tree shrews for EV-A71 neuropathogenesis. Chen et al. conducted a study in Yancheng, China (2023–2024) and identified HAdV−B3 as the dominant strain, with occasional detection of B21, C1, and C5, thereby providing valuable data for public health interventions. (Frontiers | Epidemiological characteristics of adenovirus in children in Yancheng, China, 2023-2024). At the cellular level, Zhang et al. reported the discovery of a fusion ORF3a-E-sgRNA in SARS-CoV-2, which enhances ribosomal protein S3 expression, supports viral assembly and release, and aids immune evasion. Differences in transcriptional levels may explain distinct infection processes between Wuhan and XBB strains (Frontiers | A fusion ORF3a-E subgenomic RNA involved in SARS-CoV-2 infection efficacy by influencing cellular protein synthesis). Wang et al. further elucidate viral neuropathogenesis and antibacterial immune regulation. In tree shrews, EV−A71 infection resulted in marked neurotropism and blood–brain barrier disruption, characterized by downregulation of Claudin−5 and junctional adhesion molecule A, and identified SCARB2 and Annexin A2 as potential entry receptors (Frontiers | EV-A71 invades the central nervous system and affects the blood-brain barrier in a tree shrew model). Li et al. conducted a study using a C57BL/6J mouse model of Mycobacterium abscessus infection, showing that the cardiac glycoside ouabain mitigated pulmonary inflammation by suppressing pro−inflammatory cytokine production, inhibiting NLRP3 inflammasome activation, and limiting M1 macrophage polarization, thereby suggesting therapeutic potential against drug−resistant infections. (Frontiers | Ouabain alleviates Mycobacterium abscessus-triggered inflammatory responses through dual regulation of NLRP3 inflammasome activity and M1 macrophage polarization). Collectively, these findings underscore the pathogenic roles of Claudin−5, adhesion molecules, and NLRP3, and highlight the value of diverse experimental systems in translational research.

Clinical studies in this Research Topic provide translational relevance. Using single-cell sequencing and CyTOF, researchers analyzed immune cell mobilization during viral infection and vaccination. Bodin et al. conducted a study using high-;dimensional single-;cell mass cytometry, showing that severe influenza patients have fewer regulatory MAIT and memory T/B cells but expanded inhibitory monocyte and NK cell subsets. These alterations persist into convalescence and are linked to older age and comorbidities. Through advanced single-;cell technologies, Bodin et al. provides valuable biomarkers of disease severity and informs future risk assessment and therapeutic strategies. (Frontiers | High-dimensional single-cell phenotyping unveils persistent differences in immune cell profiles between severe and moderate seasonal influenza). Wang et al. used single-;cell RNA sequencing to demonstrate that macrophages are activated early during EV-A71 infection, initiating specific immune responses closely linked to severe HFMD. They further showed that these activated macrophages not only drive persistent immune alterations but also engage pathways of neural injury and calcium signaling. Their findings highlight the central role of macrophages in EV-A71 pathogenesis.(Frontiers | Integrative scRNA-seq and transcriptomic analysis initially reveals monocyte/macrophage activation drives EV-A71-induced immune dysregulation and neural injury in severe HFMD). Martín-;Pedraza et al. conducted a prospective cohort study comparing hospitalized COVID-19 patients with uninfected healthcare workers, finding elevated plasmablasts in patients that correlated with disease severity. In contrast, healthcare workers exhibited stronger pDC and CD56^bright^ NK cell responses, suggesting protective effects. (Frontiers | Contrasting immune responses in COVID-19: insights from healthcare workers and infected patients on plasmablast, pDC, and NK cell dynamics). Ng et al. linked gut microbiota and metabolites to long-term BNT162b2 vaccine immunogenicity, showing that specific bacterial species and metabolic pathways enhance immune persistence, offering new strategies for vaccine optimization (Frontiers | Gut microbiota is associated with persistence of longer-term BNT162b2 vaccine immunogenicity).

Overall, the reviews, pathogenesis studies, and clinical investigations compiled in this Research Topic demonstrate the rapid advancement of viral immunology and vaccine development. Neutralizing antibody studies, novel vaccine approaches, and insights into type I interferon dynamics across the lifespan collectively provide a conceptual foundation for individualized immunotherapeutic strategies and more inclusive research design. Pathogenesis and infection studies have revealed the roles of key molecules such as Claudin-5, junctional adhesion molecule A, and NLRP3 in viral and bacterial infections, while expanding our understanding of pathogen pathogenic mechanisms through novel animal models such as tree shrews. Clinical investigations have employed cutting-edge technologies including single-cell sequencing, CyTOF, and metagenomics to uncover dynamic changes in immune cell populations and the profound impact of gut microbiota and metabolites on vaccine immunogenicity. Together, these advancements deepen our understanding of respiratory and enteric viral pathogenesis and point toward new opportunities for improving vaccine strategies and outcomes in vulnerable populations.

